# Quality of Life in Women with Stage 1 Stress Urinary Incontinence after Application of Conservative Treatment—A Randomized Trial

**DOI:** 10.3390/ijerph14060577

**Published:** 2017-05-30

**Authors:** Magdalena Ptak, Agnieszka Brodowska, Sylwester Ciećwież, Iwona Rotter

**Affiliations:** 1 Department of Medical Rehabilitation, Pomeranian Medical University, Szczecin, 71-210, Poland; iwona.rotter@pum.edu.pl; 2 Department of Gynaecology, Endocrinology and Gynaecologic Oncology, Pomeranian Medical University, Police, 72-010, Poland; agabrod@wp.pl (A.B.); sylciecw@sci.pum.edu.pl (S.C.)

**Keywords:** LUTS, stress urinary incontinence, perimenopause, pelvic floor

## Abstract

Stress urinary incontinence (SUI) influences quality of life in female patients. In this study, we used ICIQ LUTS QoL (The International Consultation Incontinence Questionnaire Lower Urinary Tract Symptoms quality of life) to determine the quality of life (QoL) in various domains in patients with stage 1 SUI. The study included 140 perimenopausal women subjected to urodynamic tests at the Department of Gynaecology, Endocrinology and Gynaecologic Oncology, Pomeranian Medical University, Police (Poland) in 2013–2015. The study subjects were divided into two groups, A and B. Each patient completed two questionnaires, an original survey developed by the authors and the validated ICIQ LUTS QoL. Two exercise programs, each lasting for 3 months and consisting of 4 weekly sessions, were recommended to the study subjects. The program for Group A included exercises for pelvic floor muscles (PFM) with simultaneous tension of the transverse abdominal muscle (TrA), and the program for Group B, PFM exercises without TrA tension. After completing the exercise programs, patients with stage 1 SUI, both from Group A and from Group B, showed a significant improvement in most QoL domains measured with ICIQ LUTS QoL. However, more beneficial effects of the training were observed in the group subjected to PFM exercises with TrA tension.

## 1. Introduction

In line with the definition proposed by the International Continence Society (ICS), urinary incontinence (UI) corresponds to “involuntary urination” [1,2]. Since UI affects more than 5% of general population, World Health Organization classified it as a social disease [3]. According to various sources, UI is present in 30–60% of perimenopausal and postmenopausal women, and in 70–80% of female inhabitants from nursing homes. According to the World Federation of Incontinent Patients (2012), 50% of women report incontinence at some point in their lifetime [4,5,6,7]. UI usually manifests between the 5th and 8th decade of life, and women from this age group represent 33–34% of all patients with this condition.

The most common form of UI is stress urinary incontinence (SUI), diagnosed in 50% of women with this condition [8]. SUI is defined as an uncontrolled leak of urine during some activities, such as coughing, sneezing, laughing, jumping, running or other forms of physical strain [9]. Classification of SUI based on severity of its symptoms was defined by Stamey, after Ingelman-Sandberg. Stage I SUI is defined as urinary leak during coughing, sneezing and lifting heavy objects. Stage II SUI is diagnosed in patients who perform less exhaustive forms of physical activity, such as walking and switching from sitting to standing. In stage III SUI, urinary incontinence occurs without a physical strain [9,10]. Urinary leak results from an increase in intra-abdominal pressure (IAP) and simultaneous atony of the detrusor muscle (*musculus detrusor*).

Although UI is not a life-threatening condition, due to its complex and troublesome character it may negatively affect many spheres of women’s functioning. Research on individual differences in coping with UI can be challenging. A study examining the impact of UI on the quality of life (QoL) should include functioning in mental sphere, social functions, family life, interpersonal relations, sexual contacts, business relationships and economic aspects. International Consultation Incontinence Questionnaire Lower Urinary Tract Symptoms quality of life (ICIQ LUTS QoL) is one of the questionnaires used to study QoL in patients with UI with particular reference to social effects [7]. According to the International Urogynecological Association (IUGA), ICS and International Consultation on Urological Disease (ICUD), a survey used for such purposes should be written in a clear and simple language, so it could be completed independently by all patients, irrespective of their perception level [11,12]. Questions included in ICIQ LUTS QoL refer to the influence of UI on various spheres of life: physical activity, social contacts, sexual contacts, emotional state and sleep, as well as to some specific activities and feelings related to this condition, such as wearing pads, control over fluid consumption, changing wet underwear, and discomfort associated with unpleasant odor.

ICS, IUGA, Polish Gynecological Society (PTG), Polish Urogynecological Society (PTUG), WHO, European Association of Urology (EAU) and American College of Physicians (ACP) [2,13,14] recommend physiotherapy as the first-line treatment of UI. Other recommended forms of treatment include lifestyle modification, urination at specified time intervals, behavioural therapy, pharmacotherapy and surgical management. Physiotherapy may involve physical therapy and kinesiotherapy, i.e., various exercises aimed at strengthening of pelvic floor muscles (PFM). Two biological theories explain the mechanism through which PFM training may alleviate UI. According to one theory, urethra is squeezed by its external sphincter muscle (*musculus sphincter urethral externus*), and according to another, strong PFM may support the bladder neck [15].

In 1988, Delancey [15] demonstrated that PFM tensioning leads to translocation of this muscle layer and internal organs of minor pelvis in cranioventral direction, which results in normalization of urethral positioning and in an increase in urethral pressure, consequently preventing urine leak. These observations were later confirmed by Kari Bø [16] and Thompson [17], by means of ultrasonography and magnetic resonance imaging. Miller [18] referred to the PMF-controlled translocation of the urethra before coughing as to the Knack Maneuver. This maneuver is recommended in the exercise sets for patients with SUI [19], and published evidence suggests that its application may reduce the number of urine leak incidents as early as after one week. In healthy women [20], urine leaks are prevented by spontaneous tension of PFM, which precedes the increase in intra-bladder pressure by 200–240 ms.

The second DeLancey’s theory [21] links lower incidence of SUI episodes with PFM training. According to Kari Bø, intensive training of this muscle group results in development of its mass, which provides support for minor pelvis organs [22]. This hypothesis has been recently confirmed by Pontbriand-Drolet [23]. Dynamometric studies demonstrated that PFM in women with SUI show lower tone at rest, lower maximum strength, lower contraction speed and lower endurance [23,24].

Pelvic floor muscles do not function as an independent entity. Depending on the level of physical strain, their function is supported by other synergistic muscles, among them abdominal muscles (*musculus abdominis*) and thigh adductors (*musculus adductor femoris*) [25,26,27]. Additionally, some activity of the gluteus maximus muscle (*musculus gluteus major*) was observed simultaneously to PFM tension. Synergistic activity of the transverse abdominal muscle (TrA) and PFM is explained by their common biomechanical and anatomical features, since TrA shares some fibres with the transverse perineal muscle (*musculus transversus perinei*). Activation of the TrA is a natural reflex resulting in an increase in the tension. This reflex may not be present in women after birth [28,29]. According to Sapsford [30], also other muscles, namely external oblique muscle (*musculus externus oblique*) and rectus abdominis muscle (*musculus rectus abdominis*), may recruit movement units during maximum voluntary contraction (MVC) [31].

The aim of the study was to compare the quality of life in patients with stage 1 SUI after application of a 3-month training for PFM, with concomitant tension of a synergistic muscle or without. We verified which of these two regimens is more effective in improvement of UI-specific quality of life and may constitute a model physiotherapeutic approach to stage 1 SUI.

## 2. Materials and Methods

### 2.1. Subjects

The study included 160 out of 600 women who were referred for urodynamic testing at the Department of Gynaecology, Endocrinology and Gynaecologic Oncology, Pomeranian Medical University, Police (Poland; previously, to 2016, Department of Genecology and Urogynecology) in 2013–2015. Stage 1 SUI was diagnosed on the basis of an interview with Gaudenz questionnaire [32], physical examination, and urodynamic testing. Urodynamic tests were conducted with Libra apparatus (produced in 2001, Medical Measurement System B.V. MMS, Enschede, The Netherlands).

Patients were eligible for the study if they were aged 45–60 years, had stage 1 SUI without urge incontinence, confirmed on the basis of examination with Gaudenz questionnaire and urodynamic testing with pressure-flow study, and gave their written informed consent to participate. The list of exclusion criteria included an age <45 or >60 years, a UI of different type or stage, concomitant pelvic organ prolapse, and a lack of informed consent. The abovementioned criteria were satisfied by a total of 150 subjects. Protocol of the study was granted approval from the Local Bioethics Committee at the Pomeranian Medical University (decision no. KB0012/142/13 of 30 September 2013).

### 2.2. Methods

To evaluate the effectiveness of treatment, patients with stage 1 SUI according to Ingelman-Sandberg [9,10] were assigned to Group A (*n* = 75) and Group B (*n* = 75) using simple randomization with allocations in sealed envelopes. At the baseline, patients completed two questionnaires, both available in Polish. An original survey developed by the authors was used to collect socio-demographic and medical characteristics of the study subjects, and UI-specific QoL was measured with a standardized validated scale, ICIQ-LUTS QoL (International Consultation on Incontinence Modular Questionnaire—Lower Urinary Tract Symptoms quality of life).

Subsequently, Training Regimen A was assigned to Group A and Training Regimen B was assigned to Group B. Both groups also received vaginal estrogen therapy for a period of 3 months. ICIQ LUTS QoL was completed once again after 3 months of PFM training. In Group A, 5 patients did not complete the treatment due to qualification for surgical treatment with trans-obturatory tape method (TOT). In Group B, 5 patients withdrew from the study for an unspecified reason. Eventually, the number of patients eligible for the analysis was 140—70 in Group A and 70 in Group B.

ICIQ LUTS QoL, based on The King’s Health Questionnaire (KHQ) [33], measures the influence of urinary incontinence problems on the quality of life, limitations in general, physical and mental activities, and changes in interpersonal relations and in everyday life. The instrument contains 19 questions, each measuring the impact of SUI on a 4-item scale (not at all, a bit, moderately, a lot). The global score can range between 19 and 79 points; the higher the global score, the worse the QoL in the study subject. The questionnaire omitted the VAS scale that describes the level of inconvenience of particular symptoms, as the data available at www.iciq.net is sufficient to obtain such information. The conversion of points of ICIQ LUTS QoL is carried out on the basis of the instructions provided by Hebbar [34] based on KHQ. The reliability of the applied survey methods was evaluated by calculating Cronbach’s alpha for pre- and post-treatment scores. High pre- and post-treatment values of Cronbach’s alpha (0.717 and 0.844, respectively) point to a high reliability of the scale.

The original survey developed by the authors was used to collect such characteristics of the study subjects as age, body mass index (BMI), smoking, and risk factors of SUI (Table 1).

### 2.3. Interventions

After the initial training aimed at development of appropriate PFM tension, the patients received training programs for 3 months, with 4 sessions every week. The plan for Group A included PFM exercises with additional contraction of the transverse abdominal muscle while lying on the back with flexed knees and feet on the ground. The training included 3 series with 10 repetitions and long contractions (6–8 s) corresponding to 60–70% MVC, and two series with 10 repetitions and short contractions (1–2 s) corresponding to 30–60% MVC. The tensions were correlated with exhaling. Additionally, if necessary, the Knack maneuver was recommended in the case of coughing, sneezing, or lifting a heavy object. Patients from Group B exercised according to the same protocol, but without the contraction of the transverse abdominal muscle.

All patients were estrogen therapy in form of a vaginal globule with estriol (0.5 mg, twice a week).

### 2.4. Statistical Analyses

In further analysis, continuous variables were presented as arithmetic means, standard deviations, medians, minimum, and maximum values, and qualitative variables as numbers and percentages (fractions). Normal distribution of continuous variable was verified with Shapiro–Wilk test. Statistical significance of intergroup differences in continuous and qualitative variables was verified with a Student’s *t*-test and Pearson’s chi^2^ test, respectively. Moreover, a two-way repeated measures ANOVA, time (before treatment, after treatment) x group (1. type of treatment—Group A, 2. type of treatment—Group B) was conducted for the survey data. Post-hoc analyses were conducted with Tukey’s test. Statistical significance for all tests was set at *p* < 0.05. Statistical analysis was carried out with Statistica package, v. 12.0 PL (StatSoft, Tulsa, OK, USA).

Characteristics of the study subjects, collected with the survey developed by the authors, are presented in Table 1.

No statistically significant differences were observed in terms of BMI, place of residence, declared lifestyle, menopausal status, and smoking (Table 1).

## 3. Results

Table 2 presents the results of the ANOVA (group × time) for all domains of QoL in patients with SUI, examined with the ICIQ LUTS QoL.

The evaluation of the quality of life of patients with stage 1 SUI with the use of ICIQ LUTS QoL among patients from Groups A and B after therapy showed a significant improvement in most domains. More evident improvement was observed in Group A, which means that gymnastics according to Plan A (PFM and SM) proved to be more efficient than Plan B (PFM only). The only parameter that did not change significantly in response to training was that of limitations in interpersonal relations (Q5) (Table 2).

## 4. Discussion

Stress urinary incontinence affects QoL in female patients. In this study, we used ICIQ LUTS QoL to measure the quality of life (QoL) in various domains in patients with stage 1 SUI. The study subjects underwent conservative treatment according to two regimens, pelvic floor muscle training with or without exercises for synergistic muscle.

Both the protocols of conservative treatment used in this study and the instruments used to verify the outcomes thereof, are not free from potential limitations. Although ICIQ LUTUS QoL is a standardized scale, it still determines subjective QoL. Further, the number of previous studies that analyzed the impact of PFM training on QoL in patients of SUI is limited, so our findings cannot be compared with published evidence. Previous studies analyzed changes in QoL in women practicing isolated PFM exercises at various time periods and with different frequency.

Our study showed that, irrespective of its regimen, PFM training contributed to a significant improvement of QoL in most domains. Importantly, this refers both to a three-month PFM training with exercises for the transverse abdominal muscle (Plan A) and to a 3-month isolated PFM training. However, the outcomes of training in Group A were still better than in Group B.

Since we did not find any published studies involving ICIQ LUTS QoL, we have compared our findings to those obtained with another instrument, KHQ (The King’s Health Questionnaire). According to literature, KHQ constituted the basis for ICIQ LUTS QoL, and questions included in both scales are essentially the same [33,34].

Detailed analysis demonstrated that PMF training, irrespective of its regimen, neutralized the impact of SUI on the activities of daily living, such as cleaning, shopping, professional work, and simple activities away from home (Q3). Similar findings were previously reported by Fitz et al. [35] who compared QoL in 36 women (mean age 55.2 ± 9 years) prior to and after the implementation of PFM training. Additionally, in this study, PFM training contributed to a significant improvement in all QoL domains. However, treatment regimen included in this study included solely isolated PFM exercise. Additionally, Rett et al. [36] documented beneficial effects of isolated PFM training on QoL, observed as early as after six weeks of treatment. Beneficial effects of PFM exercises (performed lying on back and sitting on a chair for 1 h a day over a 12-week period) were also reported by Nascimento-Correia [37] in a group of older women with SUI (mean age 60.87 ± 9.05 years).

SUI is an ailment with a negative impact on sport activity and traveling. An increase in intra-abdominal pressure (IAP) is observed during running, jumping, and climbing up or down the stairs. These problems are highlighted as an important limitation by women participating in QoL studies [38,39,40]. Our study demonstrated that PFM training, irrespective of its regimen, exerted positive effects on QoL in domain Q4a, i.e., physical constraints during sport activities (walking, running, gymnastics); however, more evident improvement was observed in the group participating in PFM training with additional exercises for supporting muscles.

Fitz et al. [35] reported similar findings after a 3-month PFM training used as a conservative treatment of SUI. Additionally, Rett et al. [36] documented a significant change in physical activity of women with UI, subjected to conservative treatment. However, these studies analyzed solely the effects of isolated PFM training. In the study conducted by Hirakawa et al. [41], women subjected to PFM training with biofeedback and without were followed-up twice as long as in previously mentioned experiments. Additionally, this study documented significant changes in the physical activity domain of QoL in both groups.

Our hereby presented findings imply that PFM, irrespective of its regimen, significantly improved QoL in a domain related to limitations in social contacts, visiting or meeting friends, and family relations (Q4b). These observations are not necessarily consistent with previously published data. According to Pereira [42], individualized treatment did not contribute to significant changes in this domain of QoL in a group of 17 patients. Perhaps, the duration of treatment in the latter study (6 weeks) was too short to produce any statistically significant improvement. Quite different results were reported by Rett et al. [36], who examined patients with SUI subjected to a 6-week training regimen (45 min twice a week). At the end of the study, improvement of QoL was observed in 26 subjects. Perhaps, better outcomes of PFM training documented in the latter study were associated with a large number of PFM contractions (200) and the application of biofeedback. 

Initial results of statistical analysis conducted within the framework of this study implied that conservative treatment did not exert a significant effect on QoL related to intimate relationships and sexual life (Q5). However, post-hoc analysis with Tukey’s test documented significant effects of treatment in both Group A and Group B. Fitz et al. [35] did not observe statistically significant changes in this domain of QoL in a group of patients participating in three-month training supervised by a qualified urogynecological physiotherapist. However, this study included solely women subjected to isolated PFM training. Additionally, Rett et al. [36] reported similar outcomes of conservative treatment, namely a lack of its effects on interpersonal relationships of patients with SUI. 

Domain Q6 examines the influence of SUI on QoL based on mood changes, namely the presence of depressive mood, anxiety, nervousness, and low self-esteem. Our study documented significant differences in scores for this domain, either between Group A and Group B, or between pre- and post-treatment results. PFM training with additional exercises for supporting muscle (Regimen A).

According to Coyne et al. [43,44], UI, irrespective of its type, constitutes a huge emotional burden, which, not infrequently, may lead to depression. According to literature, the prevalence of depression in patients with UI is three-fold higher than in individuals without lower urinary tract ailments [42]. In all previously mentioned studies, conducted by Fitz [35], Pereira [42], and Rett [36], isolated PFM training lasting for 6 or 12 weeks contributed to a significant improvement in this domain of QoL in patients with SUI. 

Both programs of PFM training, especially Regimen A (PFM training with additional exercises for supporting muscles) exerted a significant effect on QoL related to sleep, the presence of fatigue or exhaustion (Q7). Additionally, Fitz et al. demonstrated that a three-month training (three sessions a week) resulted in a significant improvement of QoL in this domain. A significant change in this domain was also observed by Nascimento-Correia et al. [37] after 12 group exercise sessions (1 h per week). After completing the training, participants of this study reported lesser changes in the excess of depletion of energy. 

Questions included in domain Q8 referred to wearing hygienic pads, control of fluid intake, changing underwear after urine leak, and concerns related to unpleasant body odor. While both PFM training regimens exerted statistically significant effects on QoL in this domain, the effects of Regimen A (PFM training + exercises for supporting muscles) were apparently better than the outcomes of Regimen B (isolated PFM training). Additionally, Fitz et al. [35] documented a significant improvement in this QoL domain. According to Nelas et al. [45], the negative impact of SUI on this domain in patients who did not receive support, have worse living conditions and were generally more susceptible to stress was bigger despite their greater involvement in activities mentioned above. Pereira et al. [39] demonstrated that both individual and group PFM training significantly altered patient attitude toward UI and resulted in less strict control in this area. Similar findings were also reported by Nascimento-Correia et al. [37].

Embarrassment is an important emotional problem of patients with SUI. UI is considered to be more embarrassing than other diseases, such as depression or cancer [46]. Our study with ICIQ LUTS QoL demonstrated that conservative treatment including PFM training and exercises for supporting muscles (Regimen A) contributed to a significant decrease in embarrassment level (QW), while similar effect was not observed for isolated PFM training. Thus, a question arises, why PFM training with additional exercises for supporting muscles contributed to a greater decrease in embarrassment level than isolated PFM training? In the study conducted by Wan et al. [47] in a group of Chinese women examined with ICIQ UI-SF, Social Impact Scale and Incontinence Quality-of-Life Measure, QoL decreased with the increase in UI symptom severity, social isolation and internalized shame level. Probably, an inverse mechanism was involved in our study, i.e., after conservative treatment, our patients experienced less embarrassment due to lesser shame and social isolation.

We also analyzed global ICIQ LUTS QoL scores for the study subjects. The analysis demonstrated that PMF training, either with exercises for supporting muscles (Regimen A) or without (Regimen B) resulted in a significant improvement of QoL. While information provided by a comprehensive scale with many domains, such as ICIQ LUTS QoL, is detailed enough, its global score can be easily compared with the results of other studies dealing with the problem in question. Farzinmehr et al. [48] compared the outcomes of vibration training and PFM training on QoL measured with the I-QoL questionnaire. The study showed that a 12-week PFM training contributed to significant changes in QoL. The effects of PFM training on QoL were also examined by Jahromi et al. [49] in a group of 50 Canadian women aged 60–74 years. Additionally, this study, conducted with the Questionnaire for Urinary Incontinence Diagnoses International Consultation on Incontinence Questionnaire (QUID ICIQ), demonstrated that conservative treatment contributed to a significant improvement of QoL.

## 5. Conclusions

Quality of life in patients with stage 1 stress urinary incontinence improves after conservative treatment in form of pelvic floor muscle training. Both PMF training with additional exercises for synergistic muscle (TrA) and isolated PFM training contribute to a significant improvement in QoL. Since the regimen with additional exercises for the transverse abdominal muscle was shown to be more effective, it should be considered as a primary form of intervention in future studies dealing with the problem in question.

## Figures and Tables

**Table 1:** The features of Groups A and B.

The features of group A and B		Group A *n* = 70	Group B *n* = 70	*p*
Age ($\bar{x}$+ SD, years)		53.1 ± 5.5	53.0 ± 5.7	0.813 *
BMI ($\bar{x}$+ SD, kg/m²)		27.4 ± 4.6	27.4 ± 5.0	1.0 *
Place of residence (%)	city	75.7	77.1	0.842
	village	24.3	22.9	
Physical activity (%)	sitting	12.9	18.6	0.616
	active	30.0	25.7	
	mixed	57.1	55.7	
Menopausal status (%)	premenopausal	47.1	61.4	0.09
	postmenopausal	52.9	38.6	
Smoking (%)	yes	12.9	11.4	0.8
	no	87.1	88.6	

BMI—body mass index; $\bar{x}$

—average; SD—standard deviation; *p* *—t-Student test; *p*— Pearson’s chi^2^ test

**Table 2:** A two-way analysis of variance (group × time) for the International Consultation Incontinence Questionnaire Lower Urinary Tract Symptoms quality of life (ICIQ LUTS QoL).

Score	Groups	Moment of ICIQ LUTS QoL Questionnaires Execution	$\bar{x}$	SEM	*p*^ANOVA^ and Post-Hoc Tukey’s Test
**Role limitations (Q3)**	A	before	52.6	2.6	<0.0001; Ab vs. Aa: *p* = 0.0001; Bb vs. Ba: *p* = 0.0001; Aa vs. Ba: *p* = 0.002;
		after	18.1	2.4	
	B	before	51.0	2.6	
		after	30.5	2.4	
**Physical limitations (Q4a)**	A	before	56.4	2.8	0.01; Ab vs. Aa: *p* = 0.0001; Bb vs. Ba: *p* = 0.0001; Aa vs. Ba: *p* = 0.903;
		after	19.5	2.2	
	B	before	58.6	2.8	
		after	33.6	2.2	
**Social limitations (Q4b)**	A	before	24.8	2.3	<0.0001; Ab vs. Aa: *p* < 0.0001; Bb vs. Ba: *p* < 0.0001; Aa vs. Ba: *p* = 0.0160;
		after	6.8	1.6	
	B	before	29.5	2.3	
		after	22.1	1.6	
**Relationship limitations (Q5)**	A	before	30.0	3.3	0.126; Ab vs. Aa: *p* = 0.0002; Bb vs. Ba: *p* = 0.515; Aa vs. Ba: *p* = 0.057;
		after	15.0	3.1	
	B	before	31.4	3.3	
		after	26.4	3.1	
**Emotions (Q6)**	A	before	38.3	2.4	<0.0001; Ab vs. Aa: *p* < 0.0001; Bb vs. Ba: *p* < 0.0001; Aa vs. Ba: *p* < 0.0001;
		after	11.4	2.1	
	B	before	39.0	2.4	
		after	27.9	2.1	
**Sleep/energy (Q7)**	A	before	41.2	3.0	0.052; Ab vs. Aa: *p* < 0.0001; Bb vs. Ba: *p* < 0.0001; Aa vs. Ba: *p* = 0.0007;
		after	19.8	2.3	
	B	before	47.4	3.0	
		after	34.3	2.3	
**Severity measures (Q8)**	A	before	56.6	2.6	<0.0001; Ab vs. Aa: *p* < 0.0001; Bb vs. Ba: *p* < 0.0001; Aa vs. Ba: *p* < 0.0001;
		after	24.3	2.1	
	B	before	55.8	2.6	
		after	43.0	2.1	
**Shame (QW)**	A	before	38.1	3.7	0.0001; Ab vs. Aa: *p* = 0.0001; Bb vs. Ba: *p* = 0.058; Aa vs. Ba: *p* = 0.002;
		after	14.8	2.9	
	B	before	37.6	3.7	
		after	31.4	2.9	
**Suma scores**	A	before	337.9	11.6	<0.0001; Ab vs. Aa: *p* < 0.0001; Bb vs. Ba: *p* < 0.0001; Aa vs. Ba: *p* < 0.0001;
		after	129.7	10.6	
	B	before	350.2	11.6	
		after	249.2	10.6	

“moment of ICIQ LUTS QoL questionnaires execution”: before gymnastics, after gymnastics; $\;\bar{x}$—average; SEM—standard error of the mean; Ab—scores before gymnastic in Group A; Aa—scores after gymnastic in Group A; Bb—scores before gymnastic in Group B; Ba—scores after gymnastic in Group B.
